# Clinical Characteristics of 47 Death Cases With COVID-19: A Retrospective Study at a Tertiary Center in Lahore

**DOI:** 10.7759/cureus.12039

**Published:** 2020-12-12

**Authors:** Ahmad Ussaid, Babar Riaz, Wajid Rafai, Sohail Anwar, Faisal Baig, Khurram Saleem, Farwa Pervaiz, Zaima Firdous, Shumaila A Nasir, Farrukh Iqbal

**Affiliations:** 1 Internal Medicine, University College of Medicine and Dentistry, University of Lahore Teaching Hospital, Lahore, PAK; 2 Pulmonology, University College of Medicine and Dentistry, University of Lahore Teaching Hospital, Lahore, PAK; 3 Internal Medicine, Chaudhary Muhammad Akram Teaching and Research Hospital, Lahore, PAK

**Keywords:** d-dimer, covid-19, predictors, clinical features, mortality, ards

## Abstract

Introduction

Coronavirus disease 2019 (COVID-19) presents with a wide spectrum of symptoms, ranging from patients being asymptomatic to having life-threatening acute respiratory distress syndrome (ARDS). COVID-19 emerged as a pandemic and has led to multiple causalities worldwide. A better understanding of the clinical characteristics of the COVID-19 patients and their disease course will aid in better management of these patients and hence may positively impact their outcomes as well.

Methodology

This was a retrospective observational study conducted from^ ^April 15, 2020, to^ ^August 31, 2020, after gaining institutional review board approval at the University of Lahore Teaching Hospital, Lahore, Pakistan. A total of 47 patients with severe disease who had died due to COVID-19 during this period were enrolled by the consecutive method. Patients were evaluated for their epidemiological, biochemical, clinical, and radiological features. The modified Radiographic Assessment of Lung Edema (mRALE) score was used to calculate the extent of alveolar opacities and percentages of lung involvement in chest radiographs. Furthermore, patients' management plans were also evaluated. Data were analyzed using SPSS Statistics version 23 (IBM, Armonk, NY).

Results

The mean age of the patients was 61.53 ±13.35 years. The male-to-female ratio was 2:1, and the mean BMI was 28.05 ±3.52 kg/m^2^. Diabetes was the most prevalent comorbidity among the patients (32, 68.1%), followed by hypertension (six, 12.8%), ischemic heart disease (five, 10.6%), and chronic kidney disease (four, 8.5%) respectively. The predominant symptom observed among patients was cough (95%), followed by shortness of breath (93%), fever (63%), sputum (23%), and gastrointestinal symptoms (6.4%). The mean D-dimer was 1,567.13 ±1,903.77 ng/mL, mean ferritin was 1,730.34 ±1,382.35 ng/mL, mean C-reactive protein (CRP) was 202.59 ±104.97 mg/dl, and the mean neutrophil-to-lymphocyte ratio was 10.50 ±9.58. Bilateral lung involvement was seen among 40 (85.11%) patients whereas unilateral right lung involvement was reported in three (6.38%) and unilateral left lung involvement in four (8.51%) respectively. The mean mRALE score for bilateral lung involvement was 18.78 ±4.89. The mean area radiologically involved in bilateral lung fields was 72.12 ±18.45%, followed by unilateral right lung involvement of 67.87 ±15.97%, and unilateral left lung involvement of 61.38 ±17.95% in the cohort respectively. The most common type of radiological pathology was diffuse ground-glass opacities, which was observed in 18 (38%) patients. Most patients received antibiotics (39, 63.83%), while nine (19%) received tocilizumab, four (8.5%) had antiviral therapy, and three (6.4%) were given plasma treatment. All patients received glucocorticoids and anticoagulation. The most common cause of death was ARDS, which was observed in 12 (25.5%) patients.

Conclusion

This study significantly demonstrated that most cases were males above 50 years of age with chronic medical comorbidities of diabetes, hypertension, and ischemic heart disease. COVID-19 has a predilection for multisystem involvement leading to mortality. In addition, elevated D-dimer and neutrophil-to-lymphocyte ratio may be indicative of a poor prognosis. A combination of antimicrobials had no positive impact on the outcomes in this cohort. It is difficult to predict the efficacy of tocilizumab and remdesivir as only a few patients in the cohort received these drugs.

## Introduction

A new type of coronavirus called severe acute respiratory syndrome coronavirus 2 (SARS-CoV-2) was identified in Wuhan, China, in late December 2019 following an influx of patients with pneumonia of unknown etiology. All these patients had been reported to be working at the seafood market, the epicenter of the outbreak, in Wuhan, China [1,2]. The disease that this virus causes has been named coronavirus disease 2019 (COVID-19). Coronaviruses have become a significant pathogen related to progressive respiratory sickness. This is a group of single-stranded RNA (+ssRNA) viruses that can be segregated into various species. For reasons not clearly delineated, these viruses can spread across different species, causing a different spectrum of symptoms, by not affecting one species at all, and causing extreme ailments in others, for example, the Middle East respiratory syndrome (MERS) and severe acute respiratory syndrome coronavirus (SARS-CoV) infection [3]. Although 80% of the clinical cases are mild or asymptomatic, a high level of contagiousness has made the disease worrisome for healthcare professionals across the globe. As a consequence, the World Health Organization (WHO) recognized it as a public health emergency and declared it as a pandemic in early March 2020 [4].

The median incubation period for COVID-19 is five days (range: 2-14 days). COVID-19 patients have presented with a diverse array of symptoms so far, the most prevalent being fever, cough, and shortness of breath in severe cases. Differentiating mild to moderate diseases from severe ones can help clinicians to correctly triage and manage cases [5]. Initial reports from Wuhan revealed quite a high case fatality rate for COVID-19 with a wide range of clinical presentations. In view of the mass casualties reported during SARS-CoV and MERS epidemics, severe acute respiratory syndrome coronavirus 2 (SARS-CoV-2) has proven to be equally lethal as evidenced by the worldwide case fatality rate [6].

Around 20% of COVID-19 patients develop critical illnesses, requiring management in the high-dependency or intensive care units. Severe disease has been associated with acute respiratory distress syndrome (ARDS), shock, and multi-organ dysfunction through different possible mechanisms [7,8]. There have been a few common radiological characteristics associated with the disease severity, such as deranged liver enzymes and elevated positive acute phase reactants and D-dimer. Although there is no definitive treatment modality established to date, oxygen supplementation, intravenous (IV) steroids, IV antibiotics, mechanical ventilation, and management in intensive care units have been the main options for healthcare workers in patient management [9,10]. ARDS remains a common feature of SARS associated with other viruses belonging to the same class, including SARS-CoV and MERS coronavirus (MERS-CoV) [11].

In light of the above observations, this study was aimed to develop a better understanding of the clinical characteristics of COVID-19 patients among the local population who had unfortunately died. We believe our findings will also help clinicians to flag up patients with severe disease at the earliest for a more pragmatic approach in management from the beginning itself in order to reduce mortality rates.

## Materials and methods

Medical records of 47 patients who died of COVID-19 between April 15, 2020, and August 31, 2020, were collected. The information recorded included demographics, exposure history, medical history, comorbidities, symptoms, laboratory findings, chest X-rays, clinical management, and complications (cause of death). This was a retrospective observational study conducted at the University of Lahore Teaching Hospital, Lahore, Pakistan after obtaining the institutional review board approval. Informed consent was waived off due to the retrospective nature of the study. A nonprobability consecutive sampling method was used. All patients had been tested with COVID-19 nasopharyngeal swab polymerase chain reaction (PCR) at the time of presentation, and only confirmed cases were considered for this study. Different hematological and biochemical parameters, which included a full blood count, liver function tests, renal function tests, ferritin, D-dimer, C-reactive protein (CRP), lactate dehydrogenase (LDH), creatine phosphokinase (CPK), procalcitonin (PCT), and troponin I, of the patients from their presentation to their death were evaluated. All the patients had their blood tests done at the same laboratory. The chest radiographs were evaluated for the extent of involvement of the lungs using the mRALE score by underlying pathology (0=none, 1=<25%, 2=25-50%, 3=50-75%, and 4=>75% involvement). Each lung score was then multiplied by an overall density score (1=hazy, 2=moderate, 3=dense). The sum of scores from each lung was determined to be the mRALE score. Thus, a normal chest radiograph (CXR) received a score of 0, while a CXR with complete consolidation of both lungs received the maximum score of 24. mRALE differs from the original RALE score in that the lungs are not divided into quadrants. The length of hospital and intensive care unit stay was also evaluated. The treatment types that were used to manage the patients were also recorded.

All data were analyzed using SPSS Statistics version 23 (IBM, Armonk, NY). Quantitative variables such as laboratory parameters, age, BMI, vitals, Glasgow Coma Scale (GCS), length of hospital and intensive care unit stay, and oxygen requirement were presented as means and standard deviations. Qualitative variables such as gender, comorbidities, symptoms, type of radiological pathology and pattern of lung involvement (unilateral or bilateral), type of respiratory support, and reasons of mortality along with the type of treatment were presented as frequencies and percentages. A Kaplan-Meier survival curve was also demonstrated to represent the relationship between the number of days from admission to death.

## Results

The mean age of the patients who had died due to COVID-19 was 61.53 ±13.35 years. The male-to-female ratio was 2:1, and the mean BMI was 28.05 ±3.52 Kg/m^2^. The epidemiological (age, sex) and clinical characteristics (BMI, comorbidities, symptoms and vitals, Glasgow Coma Scale) of the patients are summarized in Table 1.

**Table 1:** Epidemiological and clinical characteristics BMI: body mass index; SD: standard deviation; SOB: shortness of breath; GCS: Glasgow Coma Scale

Variables		Values
Age (years), mean ±SD		61.53 ±13.35
Sex		
Male, n (%)		29 (61.7%)
Female, n (%)		18 (38.3%)
BMI (kg/m^2^), mean ±SD		28.05 ±3.52
Comorbidities		
Diabetes, n (%)		32 (68.1%)
Hypertension, n (%)		6 (12.8%)
Ischemic heart disease, n (%)		5 (10.6%)
Chronic kidney disease, n (%)		4 (8.5%)
Symptoms on presentation		
Fever, n (%)		30 (63.8%)
Cough, n (%)		45 (95.7%)
SOB, n (%)		44 (93.6%)
Sputum, n (%)		11 (23.4%)
Gastrointestinal symptoms, n (%)		3 (6.4%)
Sick contact, n (%)		3 (6.4%)
Vitals		
Heart rate (beats per minute), mean ±SD		108 ±13.77
Respiratory rate (breaths per minute), mean ±SD		27.62 ±4.38
Oxygen saturation (%), mean ±SD		76.72 ±14.51
Oxygen requirement, mean ±SD		14.30 ±3.73
GCS score, mean ±SD		12.83 ±2.36

The hematological and biochemical laboratory parameters for all patients are presented in Table 2.

**Table 2:** Laboratory characteristics SD: standard deviation: N/L ratio: neutrophil to lymphocyte ratio; CRP: C-reactive protein; LDH: lactate dehydrogenase; ALT: alanine aminotransferase; AST: aspartate aminotransferase; CK: creatine kinase; INR: international normalized ratio

Hematological and biochemical parameters	Values (mean ±SD)
Hemoglobin (g/dL)	12.82 ±1.98
Hematocrit (%)	38.67 ±6.93
White blood cells (x 10^3/^µL)	14.34 ±7.14
Neutrophil (%)	82.06 ±8.00
Lymphocyte (%)	11.30 ±6.64
N/L ratio	10.50 ±9.58
Platelet count (x 10^3/^µL)	259.64 ±121.48
D-dimer (ng/mL)	1,567.13 ±1,903.77
Ferritin (ng/mL)	1,730.34 ±1,382.35
CRP (mg/dL)	202.59 ±104.97
LDH (mg/dL)	847.51 ±524.48
ALT (IU/L)	126.81 ±507.59
AST (IU/L)	207.74 ±932.86
Bilirubin (mg/dL)	0.65 ±0.34
Troponin I (ng/mL)	31.40 ±103.47
CK (mg/dL)	590.49 ±951.73
Pro BNP (pg/mL)	704.40 ±1,397.17
Sodium (mmol/L)	134.36 ±8.82
Potassium (mmol/L)	3.97 ±0.85
Procalcitonin (ng/mL)	3.16 ±14.4
Serum creatinine (mg/dl)	1.5 ±1.37
Prothrombin time	14.55 ±2.28
Activated thromboplastin time	38.95 ±8.55
INR	1.10 ±0.19

**Figure 1:**
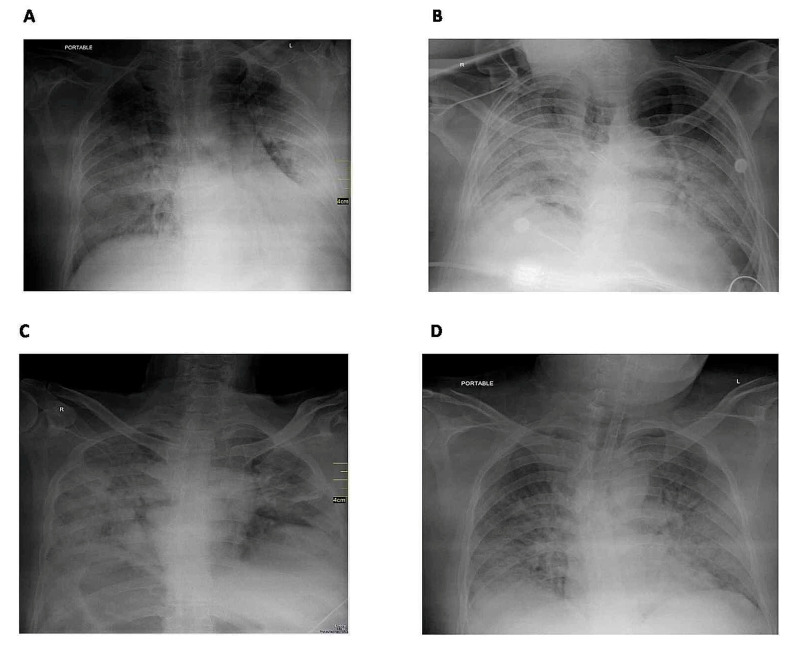
Chest radiographs A: chest radiograph of a 44-year-old male, showing bilateral peripheral, middle, and lower zone patchy consolidation with ground-glass opacities. B: chest radiograph of a 75-year-old male, showing bilateral peripheral, middle, and lower zone patchy consolidation with ground-glass opacities. C: chest radiograph of a 53-year-old male, showing bilateral peripheral, middle, and lower zone diffuse consolidation with ground-glass opacities. D: chest radiograph of a 41-year-old male, showing bilateral peripheral, middle, and lower zone patchy peripheral ground-glass opacities

Radiological characteristics including the type of alveolar opacities are presented in Figure 1 and Table 3.

**Table 3:** Radiological characteristics *Other includes reticular, hazy, and reticulonodular opacities GGO: ground-glass opacities; mRALE: modified Radiographic Assessment of Lung Edema; SD: standard deviation

Radiological characteristics	Values (mean ±SD)
mRALE score	18.78 ±4.89
Bilateral lung involvement (%)	72.12 ±18.45
Unilateral right lung area involvement (%)	67.87 ±15.97
Unilateral left lung area involvement (%)	61.38 ±17.95
Type of alveolar opacity	Frequency (%)
Diffuse GGO	18 (38.3%)
Peripheral GGO	2 (4.3%)
Basal consolidation	3 (6.4%)
Diffuse consolidation	3 (6.4%)
Patchy consolidation	13 (27.7%)
Other*	8 (17%)

**Figure 2:**
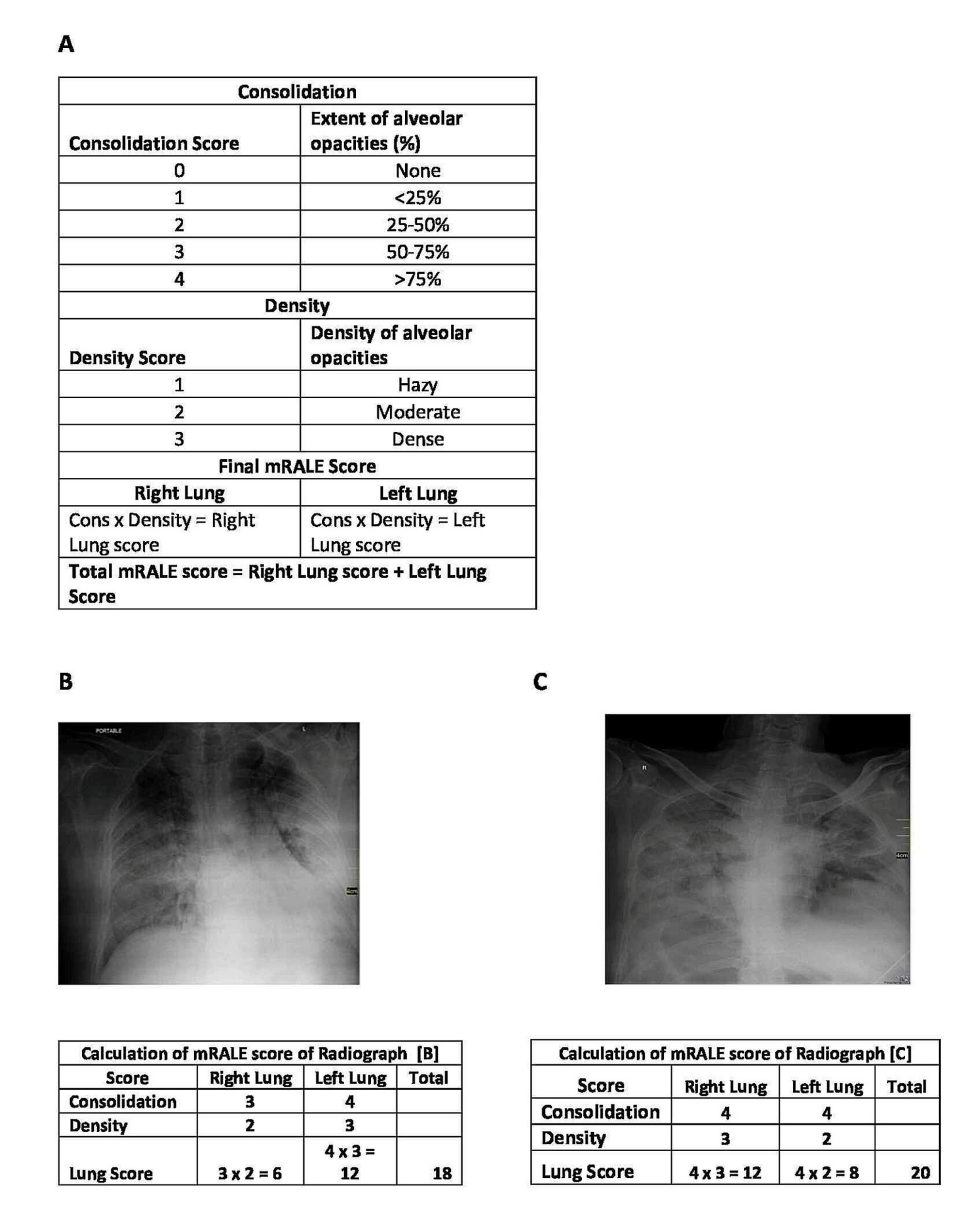
Illustration of the mRALE scoring system A: mRALE scoring system. B: calculation of mRALE score for chest radiograph B. C: calculation of mRALE score for chest radiograph C mRALE: modified Radiographic Assessment of Lung Edema

The modified Radiographic Assessment of Lung Edema (mRALE) scoring system for chest radiographs is illustrated in Figure 2.

Meropenem in combination with azithromycin was the most commonly used antibiotics regimen among patients (30, 63%), followed by a combination of antibiotics of more than three different types (nine, 19%). This was prescribed after the clinical observation of the condition of the patient and after gathering evidence of superadded bacterial infection. All 47 patients received enoxaparin and steroids as part of the standard management protocol. However, only nine (19%) patients received tocilizumab, whereas four (8.5%) patients received remdesivir, and only three (6.4%) patients received plasma infusion.

The mean fraction of inspired oxygen (FiO_2_) required was 94.68 ±18.27% for patients who required either non-invasive positive pressure ventilation (NIPPV), high-flow nasal cannula (HFNC) oxygen therapy, or mechanical ventilation. The type of airway support used, hospital and intensive care unit stay, and reasons for mortality are outlined in Table 4.

**Table 4:** Details of airway support, admission duration, and reasons for mortality ICU: intensive care unit; HFNC: high-flow nasal cannula; NIPPV: non-invasive positive pressure ventilation; ARDS: acute respiratory distress syndrome; SD: standard deviation

Variables		Values
Airway support		
Mechanical ventilation, n (%)		21 (44.68%)
HFNC, n (%)		23 (48.93%)
NIPPV, n (%)		3 (6.4%)
Length of hospital stay (days), mean ±SD		4.23 ±3.12
Length of ICU stay (days), mean ±SD		4.13 ±3.16
Reasons for mortality, n (%)	Respiratory failure	12 (25.5%)
	Septic shock	6 (12.8%)
	ARDS	12 (25.5%)
	Diabetic ketoacidosis	1 (2.1%)
	Renal failure	2 (4.3%)
	Meningoencephalitis	1 (2.1%)
	Heart failure	8 (17.02%)
	Multi-organ failure	5 (10.6%)

Mechanical ventilation was not opted for 23 (48.93%) patients who had signed Do Not Resuscitate/Do Not Ventilate (DNR/DNV) forms.

## Discussion

This retrospective study, conducted at a tertiary care center in Lahore, Pakistan, is one of the largest case series of patients who died of COVID-19 to date This study highlights the clinical characteristics of deceased patients during the first wave of COVID-19. The study included 47 patients with confirmed COVID-19 PCR test who had presented with severe disease. These patients shared some common characteristics such as older age, male predominance, higher BMI, and chronic medical comorbidities such as diabetes, hypertension, cardiovascular disease, and renal impairment. Deceased patients had a more critical disease course due to more severe hypoxemia. They had a higher tendency to develop serious complications leading to death. The experience gained in the interim and the valuable information extracted will augment the knowledge about this disease, which will help in achieving better outcomes for COVID-19 patients in future waves until we have a definitive curative or preventive treatment on board.

The mean age of the deceased patients in our study was 61.53 ±13.35 years, with a male predominance (61.7%), which is in line with a study conducted in Wuhan, China by Chen et al. [12]. This study supported our findings that seriously ill patients are likely to be in an older age group. We also found that patients who had died had high BMI, and the most commonly observed comorbidity among them was diabetes followed by hypertension, which is a new finding in comparison to previous studies [10,13-16]. This is further supported by the fact that in our study we encountered patients who had recurrent diabetic ketoacidosis that had been difficult to manage, and it added to the mortality burden.

The most notable signs and symptoms highlighted in our study were tachycardia, tachypnea, cough (94%), dyspnea (93%), fever (63%), increased oxygen demand, and disorders of consciousness. This indicates that most of the deceased patients had severe disease on presentation, and the onset of certain symptoms with alarming signs might help the treating physicians to flag up patients who are at risk of poor outcome for better care, which are findings consistent with most of the recently published studies [14-16]. A report by Du et al., which included clinical characteristics of 85 deceased patients in China, found that older age, male gender, and D-dimer of >1 mcg/ml could help in early identification of patients who may have poor outcomes [17]. Our study found that mean D-dimer levels among the deceased was 1.5 mcg/ml (1,567.13 ±1,903.77 ng/mL) and that 43% of our patients had D-dimer of >1 mcg/ml (1,000 ng/mL). Furthermore, about half of the patients who had died had procalcitonin levels of more than 1 ng/mL, which indicates a higher likelihood of superadded bacterial sepsis and death. Of note was significantly elevated cardiac troponin I (mean: 31.40 ±14.40 ng/mL) and LDH, indicating severe cardiac insult in the deceased patients, which are findings in line with previous studies [18]. We noted significantly elevated liver enzymes (ALT, AST), neutrophilia, lymphopenia, and elevated neutrophil-to-lymphocyte ratio, CRP, and ferritin levels among our cohort. All these findings cumulatively point towards the disease severity and risk of mortality; of note, deranged liver functions, lymphopenia, and elevated neutrophil-to-lymphocyte ratio were common among all patients who had died, which is consistent with previous studies [19,20].

From a practical standpoint, it is quite difficult for the doctors in personal protective equipment (PPE) to perform examinations of the patients using standard techniques such as auscultation and observation for signs of hypoxia. Hence, laboratory tests and chest radiographs have become critical tools in documenting disease severity and monitoring progress and treatment outcomes. We determined that bilateral pneumonia with a high mRALE score calculated based on the type and extent of chest radiographic involvement may also be one of the indicators of poor prognosis [21,22]. It should be noted that multiple antibiotics administration did not change the course and outcome of the disease. However, the rational use of antibiotics still remains justified. ARDS was the major complication leading to death in our series followed by cardiac complications, multi-organ failure, and septic shock, which is consistent with previous literature [14,16,18,20,23].

**Figure 3:**
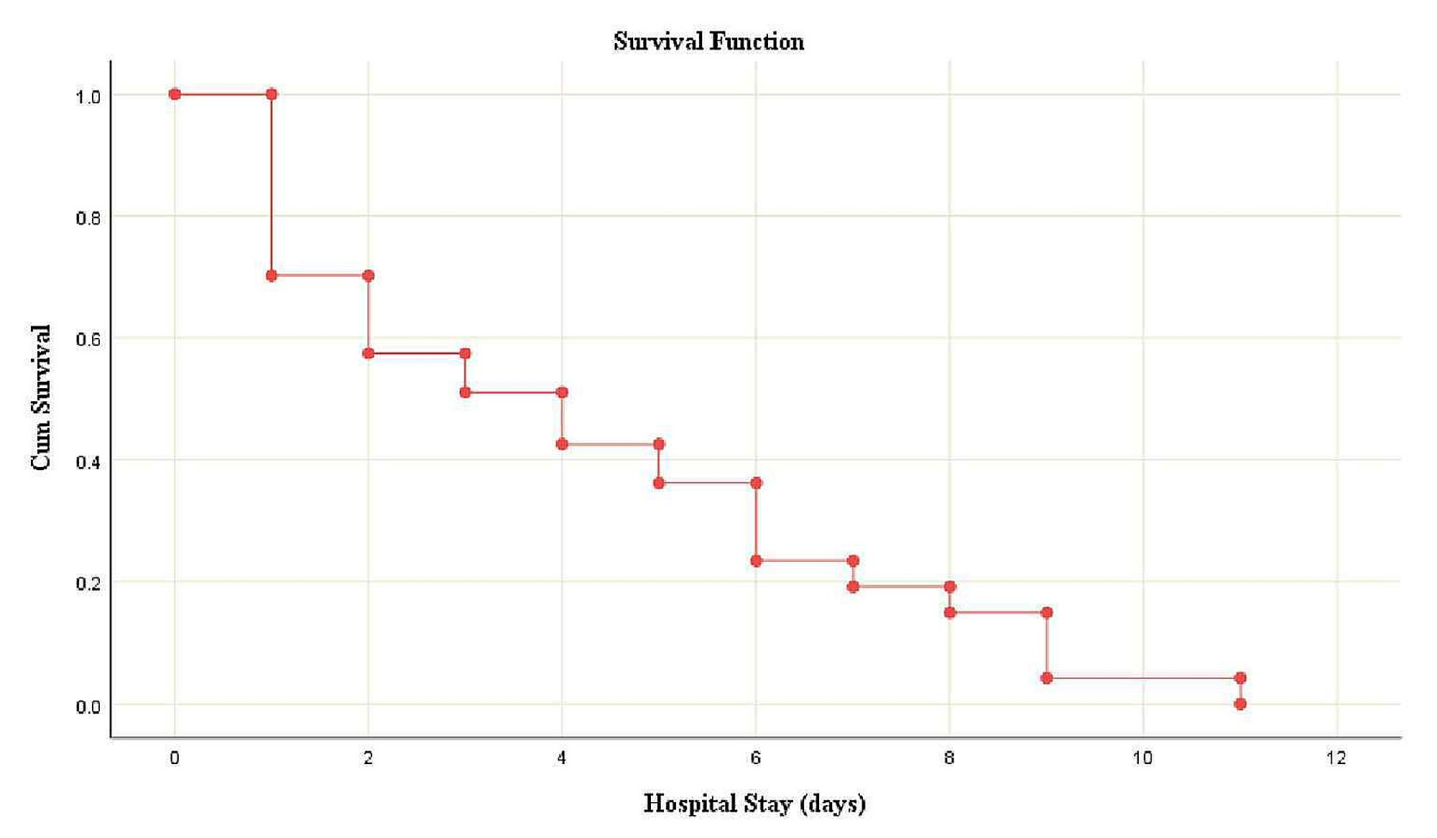
A Kaplan-Meier survival curve from hospital admission to death with coronavirus disease

Thus, advanced age (>60 years), male sex, obesity, and comorbidities, particularly diabetes with significantly elevated positive acute phase reactants and D-dimers, are believed to be significant risk factors for serious disease, requiring management in the intensive care unit, and death from SARS-CoV-2 infection. We thus hypothesize that D-dimer could be used as a prognostic marker. Hence, early vigilant monitoring with the best supportive care is required in such high-risk patients. A Kaplan-Meier survival curve is demonstrated in Figure 3.

Our study has a few limitations. Firstly, due to the retrospective nature of the study, we could not examine whether a change in the management of patients could have brought about any change in their outcomes. Secondly, we could not assess radiological disease severity via CT scans, which would have given a better understanding of the disease and helped in monitoring progress and treatment outcomes. Thirdly, only fatal cases (patients who had died) were included. A prospective study including patients with both fatal and nonfatal diseases will provide more valuable and conclusive data. Finally, only a few patients received tocilizumab and remdesivir in this series. This was due to the non-availability of these drugs, non-affordability, and delay in the availability of standard guidelines for their use during the early phase of the first-wave peak. Hence, we could not interpret the efficacy of these drugs based on such a relatively small cohort. Further prospective studies including a large number of patients will be needed to provide useful data to predict their efficacy.

## Conclusions

This study demonstrated that most patients who had died of COVID-19 were males above the age of 50 years, who had a high BMI and chronic medical comorbidities such as diabetes, hypertension, and ischemic heart disease. COVID-19 is a diverse condition given its multisystem involvement leading to multisystem complications and demise through various mechanisms. Elevated D-dimer levels, significantly high neutrophil-to-lymphocyte ratio, high mRALE score, and elevated procalcitonin levels may indicate poor prognosis. Diabetes was the most common comorbidity observed in the cohort, and this highlights the importance of improvised management strategies to achieve better glycemic control to improve patient outcomes in the future. The survival rate was inversely proportional to the length of stay in the hospital in patients with severe disease. All these alarming features in COVID-19 patients are independent risk factors and indicative of disease severity and prognostic predictability. Hence, the earlier they are recognized, the better the chances for favorable outcomes.
